# Microrobots in Micromachines

**DOI:** 10.3390/mi13081207

**Published:** 2022-07-29

**Authors:** Luigi Fortuna, Arturo Buscarino

**Affiliations:** 1 Dipartimento di Ingegneria Elettrica Elettronica e Informatica, University of Catania, 95124 Catania, CT, Italy; arturo.buscarino@unict.it; 2 IASI, Consiglio Nazionale delle Ricerche (CNR), 00185 Roma, RM, Italy

Robotics and micromachines are challenging topics in engineering. Both topics require, in fact, a multidisciplinary approach to define advanced solutions related to the design of mechanical and electronic aspects. Moreover, addressing these topics involves considering novel materials and novel power supply technologies, especially for the design of autonomous systems. In the area of micromachines devoted to micrometric positioning, the control techniques are also particularly important.

When robotics and micromachines are joined in the design of a system and, therefore, when the class of microrobots arises, the emerging problems are even more outstanding. The topics related to micromachines often offer new ideas in realizing specific robots, as classic robotic solutions related to actuators and control strategies can inspire the definition of the corresponding solutions for microrobotics. Therefore, in this scenario, twelve original contributions have been selected and discussed in this Editorial, thus covering advanced subjects of research ranging from new classes of robots to innovative sensors and timely medical applications. The readers must look at the contributions from an interdisciplinary point of view, obtaining ideas that can be used to develop new systems working at the microscales starting from macroscales solutions. Moreover, another aspect that is remarked is related to the mathematical modeling of the considered systems, which allows the conception of advanced systems in terms of size specifications and required performances.

The selected papers can be organized in organic groups. The papers [1,2,3,4,5] are related to robots for which their peculiar characteristics stand in the use of soft materials and particular actuators. These papers can inspire further classes of microrobots based on soft micromaterials, such as polymer- and microfluidic-based actuators. Indeed, microfluidics is a challenging topic in micromachinery as it offers particular perspectives in the realization of microactuators. Microfluidics is a wide area of research focusing on this timely technology and is generally used in lab-on-chip designs, but it must be employed in more fields, such as microrobotics.

Moreover, papers [6,7] focus on robots for biomechanical applications, areas of interest for future applications in walking, and remote-handling microrobots.

Therefore, the first set of papers concerns medium-sized robots but with the peculiarity that they cover more topics that can be inspiring in defining micromachine-based robots. Indeed, micromachines in a wide sense do include microrobots, as they contains sensors, actuators, and intelligent control devices. Smart materials and the innovative control of power supply systems lead to the microrobot concept. In fact, this particular class of micromachines is the subject of papers [8,9,10]: Two are related to sensing and the actuation of magnetic-based microrobots and one is related to the microgripper area. This emphasizes how microhandling represents a research area of future interest for both micromachines and microrobots.

Considering the smaller and smaller scales in micro-nanorobotic systems, paper [11] has also been selected. Moreover, looking at a systems point-of-view in micronanorobotics network control, contribution [12] has been considered. These last two contributions are selected to remark how general strong hardware techniques at small scales and wide-range optimization algorithms in large scales can be integrated for advanced functional micromachines systems. At the end of the Editorial, some additional contributions are cited.

The main features of the core of this Editorial and the specific twelve selected papers are now briefly discussed. The papers can be organized according to characterizing keywords, as reported in Table 1.

In [1] by Chen et al., the concept of soft robots is remarked, and an accurate mathematical modelling strategy is proposed. A water-based actuator is presented and a prototype with more coupled actuators is accurately studied. Both the numerical results and experimental trends obtained by a well-studied set up show the correctness of the proposed model and the efficiency of the device. The water-based actuator proposed in this contribution has a simple structure; moreover, its efficiency leads to investigating their operations on smaller-scale systems and, possibly, by using different soft materials for robots and other microfluidics-based devices.

Roozendaal et al. [2] discuss another soft robot. It consists of an adaptive 3D-shape system useful for sitting comfort. The soft term is addressed here to shape the adaptability of the system that is controlled by air fluids contained in a controlled chamber. The problem is solved by using a continuous pressure control. The authors present more prototypes. The control devices are referred to a pump with two valves. The experimental results are significant.

In both papers [1,2], the control of soft robots, intended as adaptive shape systems with fluid control, is considered. Despite the simplicity of the approach, a rigorous mathematical study for the control of such distributed systems is required. It is a challenging research problem. Moreover, a smaller size of the system can be of interest in realizing soft microrobots controlled by microfluidics devices.

In the contribution by Kim et al. [3], a robot with artificial muscular devices based on shape memory alloy (SMA) materials is proposed. The robot is devoted to walking on fluid surfaces. The soft term is here addressed for the capability of controlling the legs on a soft surface. The size of the robot is similar to that of a microrobot. A question arises: Can the movements of the robot be controlled by changing the surface physical parameters, such as the superficial tension and the fluid’s viscosity? These appealing problems are related to the transfer of energy from and to the robot without using wires and using specific equipment.

The relevant paper presented by Lee et al. [4] regards a soft robot with a thermo-pneumatic body and wireless supply system based on coupled coils. The authors propose an innovative 3D coil system that provide more efficient energy transfers, improving the efficiency of the heater. The air pressure that allows deformations; therefore, the force for microrobot movements is based on the liquid–gas transition that is more efficient when a gradient temperature in the heater device is controlled. The authors propose Polydimethylsiloxane (PDMS)-based materials for the body of the robot and a complex mechanism of injected liquids that is finalized to drive the liquid–gas phase transition. It is proved that the 3D coil system is more efficient with respect to the 2D coils and experiments show that the obtained displacements are larger, and the energy transfer’s efficiency is highly improved. The electrical control signal is of limited amplitude, about 12 V, and the frequency is of the order of 100 kHz. The results are promising and even if the technologies adopted to build the equipment are sophisticated, the performance of the microrobot allows the design of a high-performing new class of systems. It is remarked that, in some sense, since the shape of the active body is adaptively changing, from a principle point of view, this device can be seen as a soft robot, but it is quite different from the other presented soft systems.

In the contribution by Chen et al. [5], active soft-robot equipment studied for medical applications is presented . It is a flexible robot controlled with active components that are studied for colon inspection. The robot is bio-inspired; in fact, its movements are similar to that of a worm. The structure is studied in order to be adaptable to the explored environment. The size of the robot can be decreased, making similar robots suitable for exploring narrow sites, similarly to vessels. This class of robots is appealing and shows that bio-inspired structures are very efficient for practical uses. The distributed parameter model of this class of systems allows accurate modelling in unstructured environments. This will be a further research area for investigation as it is a common problem in more existing soft-robot equipment.

The paper by Bouteraa et al. [6] includes outstanding results regarding the model of a service robot for wrist rehabilitation. The authors present some numerical and experimental results of a particular structure where motion–torque control has been accurately investigated, also taking into account the processing of electromyostimulation (EMS) signals. The presented results can be taken into account for the design of microrobots to be used in precision micromanufacturing systems, where fragile and uncertain structure materials must be handled. In fact, the control strategy and the closed-loop behavior are accurately studied and appear appropriate for their extension to a wide class of torque controlled equipment in various scales.

The results reported by Zou et al. [7] characterize with accuracy an ankle mechanical system with an accurate mathematical model that is fundamental in the control of an artificial robot finalized to the rehabilitation of the ankle area of the body. Moreover, the paper invites considering the opportunity of approaching structures with a similar dynamical behavior in a smaller scale. In fact, in the area of micromachines, it is appropriate to be inspired by the results in macro-machines to propose innovative solutions. The prototype presented in [7] stimulates and addresses the researchers in designing similar systems by using 3D printing techniques and, therefore, to get the real possibility of realizing a new generation of microrobots with the same structure proposed in the paper.

The results that are presented in the contribution by Tang et al. [8] are timely and real. They are related to real microrobots made by magnetic materials. The principle for their control is very simple and includes a vision system and a network of electromagnetic devices. The authors approach a control scheme that addresses the microrobots on the correct path by using an appropriate feedback system. This can be performed if the mathematical model of the system is well established. The peculiarity of the approach is remarked, and it is the simplicity from a conceptual point of view despite the number of robots that can be controlled. The paper resembles the idea of the complexity of Buckminster Fuller: *think globally and act locally*. In view of this consideration, the following remark arises: the importance of complexity in swarming microrobots.

The study of wireless transfer energy in microrobots is approached also in the paper by Xiao et al. [9] where a high-level technological microrobot made by the combination of various materials is considered, among them are segments of Au-Ni-Au and PPY–PSS micromachines technologies conceived mainly for drug delivery. The actuators are controlled by acoustic signals at 24 kHz. The paper presents experimental results that show the suitability of the approach. The accurate dynamical characterization of the system can be important and innovative for the a priori design of performance specifications. Moreover, the presented results are introduced in the form of a review paper that contains more information for interested readers.

The very appealing subject of realizing microgripping systems is dealt with in the contribution by Kodera et al. [10]. The importance of these devices is that they work at room temperature. The termoresistive gel makes the soft part of the system rigid while the thin structures work as grippers. The gel is heated by using lasers. The microgrip size is of about 10 μm and has been conceived for handling cells of living material. Experimental results are shown and a simple mathematical model is reported in the contribution. The system can be viewed as a combination of spring-mass systems; therefore, we should immediately understand and realize it. Combining the arrays of microgripper-like systems leads to opening an impressive area in the field of micromanipulation.

The survey by Rahman et al. presented in [11] discusses various techniques for handling Single Molecules (SM). The techniques introduced are of particular interest, first of all, in the handling of fragments of DNA and RNA. The techniques relate with advanced micro-nanofluidic devices and optofluidic technologies. Moreover, these techniques are coupled with atomic force microscopy (AFM), for which its peculiarities are presented to provide the reader with a complete view on the advanced instrumentation leading to reducing the size of the system towards microrobots and paving the way for the design of nanorobots. Therefore, this contribution will stimulate, thanks to the real examples discussed therein, the area of smaller micromachines, providing the basic and advanced existing tools needed to conceive the equipment in very small scale and stimulating the rise of new concepts and new ideas oriented toward SM analyses.

The path-planning problem, which is a common problem in more classes of robots and, in particular, in robot networks, is approached by using an improved class of Genetic Algorithms in the paper proposed by Liu et al. [12]. The authors studied an improved optimization algorithm by using a parameter weight vector. The optimization problem is of impressive importance in microrobot grids, where power demand and transient responses must be minimized by taking into account precision performances. The algorithm is well tested, and more representations of its efficiency are shown in a set of representative patterns.

Some final considerations are now drawn in concluding this Editorial. Microrobotics involves the adoption of advanced technologies for their realization. Moreover, appealing 3D printers [13,14,15] appear now as a low-cost and more efficient strategy to design, realize, and test equipment in brief periods of times. The 3D printers, similarly other micromachines and microrobots, however, require accurate micropositioning. Therefore, feedback control techniques [16,17,18] must be adequate and well conceived. The realization of effective micromachines depends on the quality of their control. Soft actuators do appear in more applications and their appropriate realization is related to microfluidic technologies [18,19,20]. Microfluidics devices require both external energy transfer and direct energy transfer by using micropumps that are, indeed, micromachines. The impressive theme of microrobots, therefore, includes a multidisciplinary approach and, in some sense, a circular point of view. Materials, sensors, control, and actuators must often be viewed not independently of each other. Micromanufacturing systems cannot be viewed independently from the particular microsystem to be realized, as they can also be a part of the whole.

## Figures and Tables

**Table 1:** Correlation matrix of the selected keywords.

	[1]	[2]	[3]	[4]	[5]	[6]	[7]	[8]	[9]	[10]	[11]	[12]
Sensors	X	X	X	X		X		X				
Actuators	X	X	X	X	X	X	X	X	X	X		
Feedback	X	X	X			X		X		X		
Advanced Techniques			X	X				X	X	X	X	
System Engineering	X	X	X	X		X		X			X	X
Computer-Aided-Design	X	X		X		X	X	X		X	X	X
Advanced Characterization				X					X		X	
Bio-inspired solutions			X		X	X	X				X	X
